# Elemental Spatial and Temporal Association Formation in Left Temporal Lobe Epilepsy

**DOI:** 10.1371/journal.pone.0100891

**Published:** 2014-06-30

**Authors:** Christopher F. A. Benjamin, Michael M. Saling, Amanda G. Wood, David C. Reutens

**Affiliations:** 1 Department of Psychiatry, University of California Los Angeles Semel Institute, Los Angeles, California, United States of America, and Computational Radiology Laboratory, Harvard Medical School, Boston, Massachusetts, United States of America; 2 Melbourne School of Psychological Sciences, The University of Melbourne, Melbourne, Victoria, Australia; 3 Department of Psychology, University of Birmingham, Birmingham, England, and Southern Clinical School, Monash University, Clayton, Australia; 4 Centre for Advanced Imaging, The University of Queensland, Brisbane, Queensland, Australia; Hospital for Sick Children, Canada

## Abstract

The mesial temporal lobe (MTL) is typically understood as a memory structure in clinical settings, with the *sine qua non* of MTL damage in epilepsy being memory impairment. Recent models, however, understand memory as one of a number of higher cognitive functions that recruit the MTL through their reliance on more fundamental processes, such as “self-projection” or “association formation”. We examined how damage to the left MTL influences these fundamental processes through the encoding of elemental spatial and temporal associations. We used a novel fMRI task to image the encoding of simple visual stimuli, either rich or impoverished, in spatial or spatial plus temporal information. Participants included 14 typical adults (36.4 years, sd. 10.5 years) and 14 patients with left mesial temporal lobe damage as evidenced by a clinical diagnosis of left temporal lobe epilepsy (TLE) and left MTL impairment on imaging (34.3 years, sd. 6.6 years). In-scanner behavioral performance was equivalent across groups. In the typical group whole-brain analysis revealed highly significant bilateral parahippocampal activation (right > left) during spatial associative processing and left hippocampal/parahippocampal deactivation in joint spatial-temporal associative processing. In the left TLE group identical analyses indicated patients used MTL structures contralateral to the seizure focus differently and relied on extra-MTL regions to a greater extent. These results are consistent with the notion that epileptogenic MTL damage is followed by reorganization of networks underlying elemental associative processes. In addition, they provide further evidence that task-related fMRI deactivation can meaningfully index brain function. The implications of these findings for clinical and cognitive neuropsychological models of MTL function in TLE are discussed.

## Introduction

The relationship between mesial temporal lobe (MTL) damage and memory impairment is fundamentally accepted in neuropsychology. Significant evidence supports a central role for the MTL in episodic memory in particular, the system supporting our ability to recreate and relive the events of our daily lives [1]–[2]. The defining characteristics of these memories include the temporal-spatial relations among their components [1], a subjective sense of the self and time, and the form of ‘autonoetic’ consciousness that allows us to mentally experience and relive events [2]. Destruction of the hippocampi early in development selectively impairs the ability to form such memories while leaving formation of other forms of memory largely intact [3].

The precise nature of the core processes impaired by MTL damage that manifest as memory impairment is a source of ongoing debate. The production of spatial and temporal associations in episodic memory has led a number of authors to argue association formation may constitute a cognitive endophenotype of MTL function (e.g., [4]). Indeed, tasks requiring creation of associations (e.g., between unrelated pairs of words) are uniquely sensitive to mesial temporal lobe damage in epilepsy [5]–[7]. This fact, together with a model postulating differing contributions for left and right MTLs in verbal and nonverbal memory respectively; i.e. “material specificity” [8]–[9], continues to form a central tenet of clinical neuropsychological assessment for surgical planning in epilepsy in many centers.

Models developed from this perspective have evolved to consider MTL substructures as processing associations in a complementary and hierarchical manner [4] [10]–[11]. Broadly, such models suggest that after information has been perceived and associated to form a perceptual or cognitive ‘item’ (‘unitization’, likely supported by extra-MTL structures), the perirhinal cortex is engaged to form or store item level associations [12]. Parahippocampal cortex then forms fixed (e.g. egocentric spatial) representations (though see also [13]), while the hippocampus allows these associations to be flexibly re-expressed in different ways [4] [13]. Significant work has now also suggested the hippocampus is central in associating information even over the very short-term (for instance, in working memory and perception; see [15] for an extensive review).

In the cognitive neuropsychological literature, a number of researchers have argued that the MTL's engagement in tasks beyond episodic memory must influence our understanding of MTL function. One model considers projection of the self into a novel context (“self projection”) as a core process in tasks engaging the MTL and a network of related brain regions [16]. Consistent with this are the findings that bilateral hippocampal damage results in impairment of both episodic memory and other cognitive domains that share the MTL network, such as topographical memory [17], and that amnesiogenic MTL damage impairs the ability to imagine new experiences [18]. Cognitively, each of these processes can be considered to require associative processing to locate the self in a novel, constructed environment. Of relevance, Spreng, Marr and Kim [19] recently compared the brain regions activated in these and related processes, namely autobiographical memory, navigation, theory of mind, and the default mode network, which are also thought to be involved in associative processing at rest (e.g., [20]) They found common engagement of the mesial temporal lobe, posterior cingulate, precuneus, temporo-parietal junction and retrosplenial cortex. The single point of highest correspondence between these networks fell within the left parahippocampal cortex [19].

The aim of this study was to examine whether the deficits in episodic memory and conjoint associatively-based functions [17]–[20], which can be affected by left temporal lobe epilepsy (TLE), might be more parsimoniously understood as the result of deficits in a more elemental process, the formation of spatial and temporal associations. These processes are fundamental to episodic memory [1]–[2], and are necessary if life events are to be replayed or relived in a spatial and temporal context. Arguably, spatial and temporal associations are also fundamental in other higher-order cognitive skills such as navigation, where spatial and temporal relationships are continually re-expressed, and theory of mind, where others' (spatially and temporally organized) experience is simulated (e.g., [21], see also [19]).

A number of studies have sought to understand which brain regions are engaged in the formation of spatial and temporal associations, typically using complex, naturalistic stimuli in healthy adults. Studies of spatial associative processing in typical adults suggest recruitment of parahippocampal cortex [22]–[24] or hippocampus proper [25]. Temporal associative processing activates left [26] and right parahippocampal cortex [27]–[28], as well as bilateral parahippocampal cortex and right hippocampus [29]. These studies have used complex and varied stimuli such as comics [26], virtual reality [27], images from participants' experience [28] and movies [29]. Complex stimuli are compelling because they are naturalistic, but because of their inherent complexity, they are difficult to accommodate in a subtraction design. In a recent study Zeidman and colleagues [30] examined spatial and temporal association formation at a more basic level by examining the neural response to dot fields in which the dots were either exponentially distributed to connote a sense of space, in the form of a vanishing horizon, or were randomly distributed to form a non-spatial percept. Spatial frequency was manipulated for spatial and non-spatial stimuli. For the low frequency condition, left parahippocampal cortex showed a greater response to non-spatial than to spatial stimuli. In the high frequency condition, by contrast, right parahippocampal cortex responded more to spatial than to non-spatial stimuli. This was taken to suggest that coding for elemental space might be a core function of parahippocampal cortex. We also chose to use basic configurations (squares and patterns) to investigate spatial and temporal associative processing in patients with left mesial temporal damage at a more fundamental level than in prior research.

Beyond our aim of examining whether deficits in higher-order cognitive skills (e.g., episodic memory) may be a function of deficits in the elemental cognitive processes of spatial and temporal association formation, we hypothesized elemental spatial and concurrent spatial-temporal processing would engage the parahippocampal cortex in typical adults. In patients with damage to a hub of the memory network, the left MTL (e.g., [19], [31]–[32]), we hypothesized that spatial and temporal information would be encoded from moment-to-moment but that the neural correlates of this process would differ from typical adults.

## Materials and Methods

### Participants

Participants included a total of 14 control participants (mean age 36.4 years, s.d. 10.5 years; 8 female; 13 right-handed) and 14 left mesial TLE patients (mean age 34.3 years, s.d. 6.6 years; 7 female; twelve right-handed); final imaging data included 14 controls and, for the patient sample, 13 (spatial) and 11 (spatial-temporal) participants (see Imaging, below). All patients had a clinical diagnosis and imaging evidence of left TLE. The majority (twelve) had structural evidence of left mesial temporal pathology on MRI or CT and eight had hippocampal sclerosis or atrophy (Table 1). One patient had a prior limited resection of a mesial temporal dysembryoplastic neuroepithelial tumor (DNET, 9 years prior); post-operative MRI demonstrated preservation of key MTL structures. Participants were recruited through Monash Medical Centre and Austin Health (Melbourne, Australia), and advertisements in local news sources. Participants were screened to rule out other neurological disorders, color blindness and significant uncorrected visual impairment.

**Table 1:** Left TLE characteristics on imaging.

ID	Structural imaging		Functional Imaging	Further detail
	Laterality on MRI	Hippocampal Involvement	EEG findings	(1) PET (2) SPECT	
P1	Left	HS	Left TL spikes	(1) Left TL decrease; (2b) MTL decrease	Also L anterior temporal cortical thickening (MRI)
P2	Left	HS	–	–	–
P3	–	–	Left frontal-anterior temporal spikes	–	–
P4	Left	HS	Left TL changes	(1) Left TL decrease	–
P5	Left	HS	Left ATL spikes	(1) Left TL decrease (medial & lateral)	Left superior middle & inferior TL gyral dysplasia (MRI)
P6	Left	No; bone defect	Left ATL spikes	(1) Left ATL decrease	Left TL pole encephalocele (MRI, CT)
P7	Left	HS	Left mid-TL theta/delta activity	–	–
P8	Left	HS	Left hemisphere theta/delta activity	–	–
P9	Left	No	Left TL spikes	(1) Left TL decrease (anterior & medial)	Prior left AT lobectomy, post-operatively L hippocampus remains normal
P10	NAD	No	Left TL spikes	(1) Left MTL decrease	Clear VEEG evidence of left TL seizure focus.
P11	Left	HS	Left fronto-temporal spikes	–	subtle hippocampal asymmetry (MRI)
P12	Left	HS	Left fronto-temporal spikes	–	–
P13	Left	No; pole thinned	Left TL spikes	(1, 2a, 2b) Left TL decrease	Left TL polar encephalocele (MRI)
P14	Left	HS	Left ATL spikes	(1) Left TL (medial, anterior) and temporo-occipital decrease	Enlarged left amygdala (MRI)

HS: hippocampal sclerosis (unilateral and congruent with laterality of MRI evidence unless otherwise noted). TL: temporal lobe. ATL: anterior temporal lobe. AT: anterior temporal. MTL: mesial temporal lobe. — indicates no data available. Available ictal and interictal EEG (electroncephalography) and VEEG (video EEG) data are presented. *2a: Ictal SPECT. 2b:Interictal SPECT.

Groups' task-related cognitive skills were characterized using the (Australian) Wechsler Adult Intelligence Scale III (WAIS-III) and Rey Auditory Verbal Learning Task (RAVLT). Two sample t-tests confirmed they were equivalent on measures of processing speed, working memory and nonverbal intelligence, but in keeping with the patients' pathology the groups differed on estimated verbal intelligence. Specifically, mean Digit Span (working memory) performance for controls was 12.50 (High Average; s.d. 3.5) and for patients was 10.57 (Average; s.d. 4.1); t(_24_) = −1.29, p = 0.21, n.s. On Digit-Symbol Coding (processing speed), controls scored 12.92 (High Average; s.d. 2.3) and patients 9.38 (Average; s.d. 3.0); t(_21_) = −1.82, p = 0.083, n.s. Estimated global nonverbal intellectual function (WAIS-III Matrix Reasoning) for controls was 13.25 (High Average; s.d. 0.9) and for patients was 12.00 (High Average; s.d. 2.6) t(_14.9_) = −1.67, p = 0.116, n.s.; estimated verbal intelligence (WAIS-III Vocabulary) for controls was 14.83 (High Average; s.d. 3.0) and for patients was 10.54 (Average; s.d. 3.9) t_(22.4)_ = 3.09; p = 0.005. Estimated verbal memory was within the normal range immediately (controls Z = 0.48, Average s.d. 1.1), patients Z = 0.01 (Average, s.d. 0.9; t(_21.4_) = 1.14, p = 0.265, n.s.) and over a short delay (post interference; controls Z = 0.40, Average, s.d. 1.1); patients Z = 0.24 Average, s.d. 1.1; t(_21.3_) = 1.39, p = 0.180, n.s. Patients performed worse at 20 minutes' delay (controls Z = 0.69, High Average; s.d. 0.7); patients Z = −0.22 (Average; s.d. 0.7); t_(20.7)_ = 2.97; p = 0.007. Neuropsychological data were available for all but one patient (Vocabulary, Digit Symbol, Matrix Reasoning) and all but two controls.

### fMRI Task

#### Scanner task

**Figure 1:**
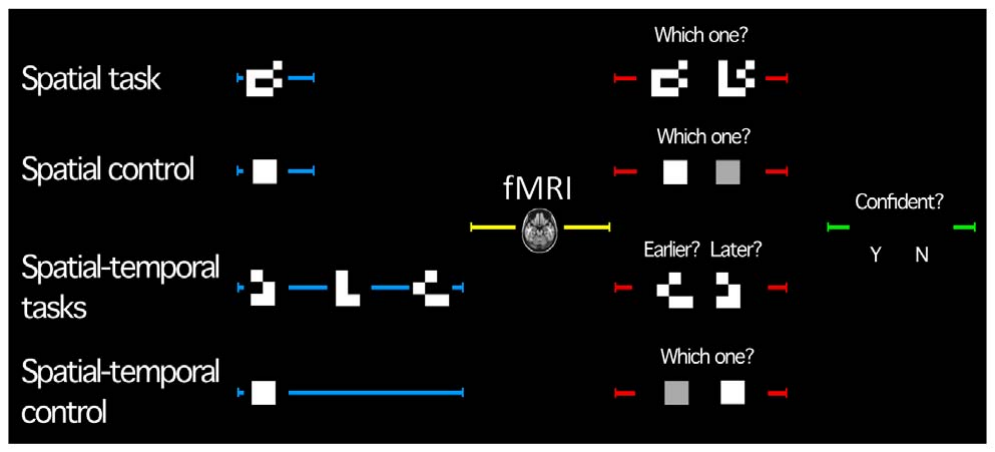
Trial structure by condition. Trials began with a perception phase (blue; contrasted in analysis). Image acquisition was time-locked to the BOLD peak from stimulus perception (image of brain). Participants then answered a question to assess whether they had attended to the stimuli (decision phase; red). Two variants of the spatial-temporal task were completed; one in which the ‘earlier’ of two images was selected, the other, the ‘later’. Decision confidence was then assessed (green). Timing was matched across each phase (control conditions).

Encoding of the elemental associations between perceptual stimuli was imaged using a subsequent memory paradigm with event-related fMRI. Sparse imaging was used due to scanner constraints; this approach restricted the number of images acquired (100). Imaging was locked to the BOLD changes following task-related neural activity; i.e., imaging centered on activity during the perception of stimuli and spatial/spatial-temporal information, occurring when the hemodynamic response function (HRF) peaked six seconds after the mid-point of stimulus perception (Figure 1). Imaging was jittered +/− 500 ms and 1000 ms around this peak [33] during which time no stimuli were presented.

#### Conditions

Five conditions were imaged: (1) a spatial task and (2) matched control condition, (3) spatial-temporal ‘early’ and (4) ‘late’ tasks with a (5) matched control (Figure 1). As piloting indicated patients at times had difficulty recalling the task at hand, each condition was presented in a single scanner run so that instructions could be presented orally and visually and comprehension confirmed. Runs (i.e., each condition) consisted of 20 trials of a single task/control type and run/condition order was counterbalanced. Accurate trials were contrasted in analysis. Conditions were designed around two subtraction analyses. The spatial control required encoding of visual luminance information (white square) presented for a given duration of time; the spatial task, encoding of the same information re-organized in a spatial pattern (white checkerboard). The spatial-temporal control was the same as the spatial control (white square), though with a different duration. The spatial-temporal task incorporated spatial information (spatially-organized checkerboards) and temporal information (three consecutive images) (Figure 1) presented for the same total duration as the control.

#### Trial structure

All task and control trials had the same structure, being 15.75 s and 18 s long for spatial and spatial-temporal runs respectively (Table 1). In each trial participants first perceived a stimulus or stimuli (Table 1, blue; 0.75 s duration spatial, 2.25 s spatial-temporal). This encoding phase was later contrasted in analysis. Image acquisition then occurred (blank screen; 8.625 s duration spatial, 7.875 s spatial-temporal). To ensure participants perceived stimuli, in a subsequent decision phase participants used boxes with single buttons held in their left and right hands to make a test response (Figure 1, red; 2 s; response laterality was pseudo-randomized) followed by a prompt to evaluate decision confidence (Figure 1, green; 2 s; confidence data not presented here). Total trial length was 15.75 ms (spatial conditions) and 18 s (spatial-temporal conditions). At the completion of imaging participants answered a debriefing questionnaire, which included questions to confirm they had completed the task as intended. Runs were counterbalanced; task order was pseudo-randomized with active tasks and controls alternating.

Importantly, BOLD signal associated with encoding of stimuli that were later remembered was compared. This was achieved using behavioral data to separate trials where subsequent memory was accurate or inaccurate. The regressor mapping inaccurate trials was ignored (weighted 0) in statistical contrasts.

#### Stimuli

**Figure 2:**
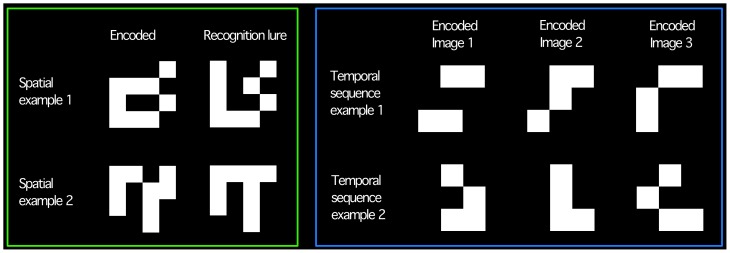
Illustrative task stimuli. *Green box*: Spatial stimuli. Imaging occurred during perception of stimuli on left, matched lures, used to confirm participants perceived the stimuli, are presented on the right. *Blue box*: Spatial-temporal stimuli (‘early’ or ‘late’ tasks). Each stimulus was presented on screen individually in sequence (left to right). Two stimuli from the sequence were then re-presented (decision phase); participants selected which of the two had been presented later or earlier.

Spatial stimuli constituted nine white and seven black patches on a 4×4 grid with a black background (Figure 2, green box). These were arranged randomly in spatial relation to one another. In the ‘decision’ phase a lure, in which the arrangement of white patches differed in a single stimulus quadrant, was presented alongside the encoded stimulus. In the spatial control the same white squares were organized into a single 3×3 square in the same 4×4 array (Figure 1) to match perceptual information other than spatial complexity. In the control decision phase, participants indicated whether they had viewed the presented stimulus or a square that was 20%, 29%, 60% or 85% of the presented stimulus's luminance. The spatial-temporal stimuli constituted four white patches and 5 black patches arranged in a random pattern within a 3×3 matrix (Figure 2, blue box). Consecutive stimuli differed in the arrangement of squares within a single (differing) quadrant of the stimulus (location of differing quadrant was pseudo-randomized). In the decision phase two stimuli from the sequence were re-presented and the participant indicated which had been presented earlier or later in sequence.

Stimuli were programmed in MATLAB [34] and presented through ‘Presentation’ [35] using a Dell Inspiron (2 Ghz P4) and Sony VPL ES1 projector. Stimulus luminance was calibrated using a Minolta Chroma meter CS-100A spot photometer. Stimulus presentation was synchronized by trial. Specific trial parameters were derived through pilot testing in university students in which image duration (250, 500, 750, 1000, 1250 ms), sequence length (3–6 images) and spatial complexity (white squares arranged on 3×3 to 9×9 grids) were manipulated to arrive at parameters where typical adults achieved approximately 75% accuracy. These parameters were then piloted and modified in two patients with TLE. Behavioral data on the final task version are presented below.

### Imaging

Imaging was completed on a 1.5 Tesla Siemens Magnetom scanner (Monash Medical Centre, Victoria, Australia). Whole brain T1-weighted images comprising 144 contiguous slices (1×1×1 mm) were acquired with a TR of 2070 ms, TE 3.93 ms, flip angle 15°, 256×256 matrix and FOV 250 mm^2^. Whole brain functional images were acquired in a sparse design due to scanner constraints and included 21 contiguous slices acquired in an interleaved manner, with voxel dimensions 2.15*2.15*7 mm, a TE of 88 ms, 90° flip angle, 128×128 matrix, FOV 250 mm^2^. TR was 15.75 s (spatial conditions) or 18000 ms (spatial-temporal); both with TA 3.94 s. Images were aligned with the longitudinal axis of the hippocampus. Data was available for the 14 controls. For TLE patients, data were available for 13 spatial and 11 spatial-temporal conditions. This was due to post-scan debriefing indicating that one patient had not performed the task correctly, and hardware/software malfunction (3 cases). Separately, participants also completed separate functional runs for tasks not forming part of this study.

### Procedure

The study was approved by the Human Research Ethics Committees at Monash Medical Centre and Austin Health in Melbourne, Australia. The approved consent process included providing participants with a plain language statement detailing the study and allowing them time to review this; a discussion where researchers answered participants' questions; and then their formally signing a copy of the consent form. Participants then completed behavioral testing and imaging over 1–3 sessions. Prior to imaging, behavioral testing involved participants completing neuropsychological assessment (above) and a practice session of the task with different stimuli.

### Analysis

#### Behavioral data

Group differences in performance were compared using Bonferroni-corrected two sample t-tests assuming unequal variance. Imaging data: Analysis was completed in SPM5 (www.fil.ion.ucl.ac.uk/spm/) with the general linear model. The first image from each functional set was removed (B0 field effects), T2* images were realigned to the first image (6 parameter rigid body transform); resliced; slice-time corrected (middle slice). The T1 was coregistered to T2* space and segmented. Images were then normalized (MNI-152 space); resampled (2×2×2 mm); and smoothed (12 mm isotropic Gaussian kernel). Correct and incorrect trials were considered separately (onsets/durations convolved with the canonical HRF) and modeled using the general linear model (event-related design). In four instances a single trial from a condition was not presented due to computer error (regressors altered accordingly). Contrasts compared task and control activation from accurate trials. Spatial-temporal ‘early’ and ‘late’ task runs were weighted equally against the single spatial-temporal control. As noted, setting task difficulty at an appropriate level meant stimulus duration was matched between tasks and their controls, but not between tasks (spatial: 750 ms; spatial-temporal: 2250 ms), so that a direct spatial v spatial-temporal task contrast was not feasible. Random effects models were generated using contrast images. Whole brain analyses were thresholded at p<0.005 uncorrected (voxel-wise) with a 5 voxel extent threshold. Coordinates reported in text are in MNI space and were labeled via the Talairach daemon (using icbm_spm2tal.m; http://brainmap.org/icbm2tal/) and visual inspection.

Reported results are cluster-wise corrected (SPM5) with reference to Gaussian Random Field theory. Where informative uncorrected voxelwise results are noted and labeled p_(uncorrected)_.

## Results

### Behavioral data

In scanner, the groups performed equivalently in all tasks. In the spatial task and its control task, control subjects performed at 95% and 96% accuracy and the TLE group at 93% and 85% accuracy respectively (spatial task: t_(24.62)_ = 0.82, p = 0.421; spatial control: t_(13.89)_ = 1.97, p = 0.070). On the spatial-temporal early, late and control tasks, the control group performed at 78%, 85% and 98% accuracy and the TLE group at 71%, 77% and 97% accuracy respectively (spatial-temporal early: t_(18.22)_ = 1.14, p = 0.269; late: t_(22.95)_ = 1.64, p = 0.115; control: t_(22.14)_ = 0.41, p = 0.687).

### Imaging data

**Figure 3:**
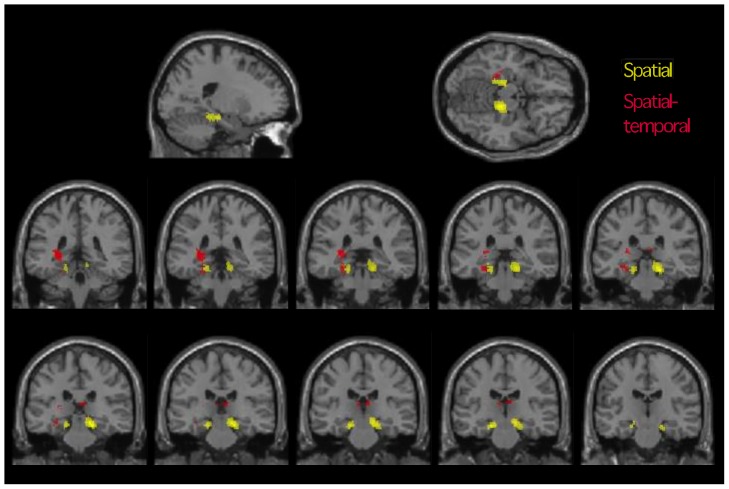
fMRI results, health controls. Spatial activation (task > control; yellow) and spatial-temporal deactivation (task < control; red) (separate analyses overlaid for comparison). Top row: Axial and sagittal slices (image coordinates −20, −38, −14). Middle and bottom rows: 2 mm coronal slices from −20, −38, −14 (top left) to −20, −20, −14 (bottom right). Clusters significant at whole-brain level. Spatial-temporal cluster extends slightly more posteriorly and extends into the fimbria/abuts the thalamus. Images masked to show mesial temporal/subcortical (de)activations of interest. Right of image is right hemisphere, p<0.005, 5 voxel threshold.

Spatial task-related activation (spatial task > spatial control) revealed foci of greater activity in the right and left posterior parahippocampal regions (Table 2; Figure 3, yellow). Of note, these activations were relatively anterior within the posterior parahippocampal region. A number of areas were deactivated during the spatial task (spatial task < spatial control), including the left middle frontal gyrus, and right and left anterior cingulate regions (Table 2). Further cluster-significant deactivations were apparent within the left middle and superior temporal gyri, the right superior temporal gyrus, left inferior parietal lobule and right postcentral and lingual gyri. The spatial-temporal condition was not associated with significant task-specific activation. A single, highly significant task-related deactivation (spatial-temporal tasks < spatial-temporal control) was apparent, however, with multiple subpeaks through the left posterior hippocampal and parahippocampal cortex (Table 2; Figure 3, red). This deactivation fell posterior to that revealed by the spatial contrast.

**Table 2:** fMRI results, controls.

P (cluster corrected)	K (cluster)	T (voxel)	Z (voxel)	Main cluster peak: MNI coords. X, Y, Z	Region
**Spatial task-related activation** *(13DOF)*					
0.001	321	5.69	3.96	18 −28 −16	Right parahippocampal region
0.043	163	5.13	3.73	−18 −22 −16	Left parahippocampal region
**Spatial task-related deactivation** *(13DOF)*					
0.000	1017	7.17	4.49	32 −28 48	Right post-central gyrus
0.000	955	6.53	4.27	−46 −28 62	Left inferior parietal lobule
0.000	847	6.38	4.22	−28 −62 28	Left middle temporal gyrus
0.004	249	6.18	4.15	24 −60 0	Right lingual gyrus
0.002	285	6.13	4.13	−44 46 16	Left middle frontal gyrus
0.000	385	6.09	4.12	−54 −40 8	Left superior temporal gyrus
0.000	727	5.92	4.05	12 8 44	Right anterior cingulate
0.000	818	5.9	4.04	66 −38 22	Right superior temporal gyrus
0.001	290	4.44	3.4	−4 −24 36	Left anterior cingulate
**Spatial-temporal task-related deactivation** *(13DOF)*					
0.011	317	5.02	3.68	−28 −34 6	Left hippocampus (posterior)

Activation contrast: task(s) > matched control. Deactivation contrast: matched control > task(s). Cluster correction per SPM5; K denotes main cluster size (sub-cluster peaks not reported).

Given the finding of opposing spatial task-related activation and spatial-temporal deactivation, the above contrast was re-run explicitly masking out voxels where activation was elevated in the spatial-temporal control run as compared with the spatial control run. This ensured the results were not a function of differing baseline magnetization between the separate runs containing the control conditions, even though this was considered unlikely due to counterbalancing of run/condition order across participants. The MTL cluster remained (t_(13)_ = 5.02; p = 0.012). Similarly, after masking the spatial condition, the right MTL cluster remained significant (t_(13)_ = 5.69, p = 0.001) and while the left was not significant with correction it was at a more lenient threshold (t_(13)_ = 5.13, p_(uncorrected)_ = 0.000). The relationship between the spatial activation and spatial-temporal deactivation was also examined; masking the spatial-temporal deactivation with the results of the spatial activation revealed only two overlapping voxels which fell within the left posterior parahippocampal region.

**Figure 4:**
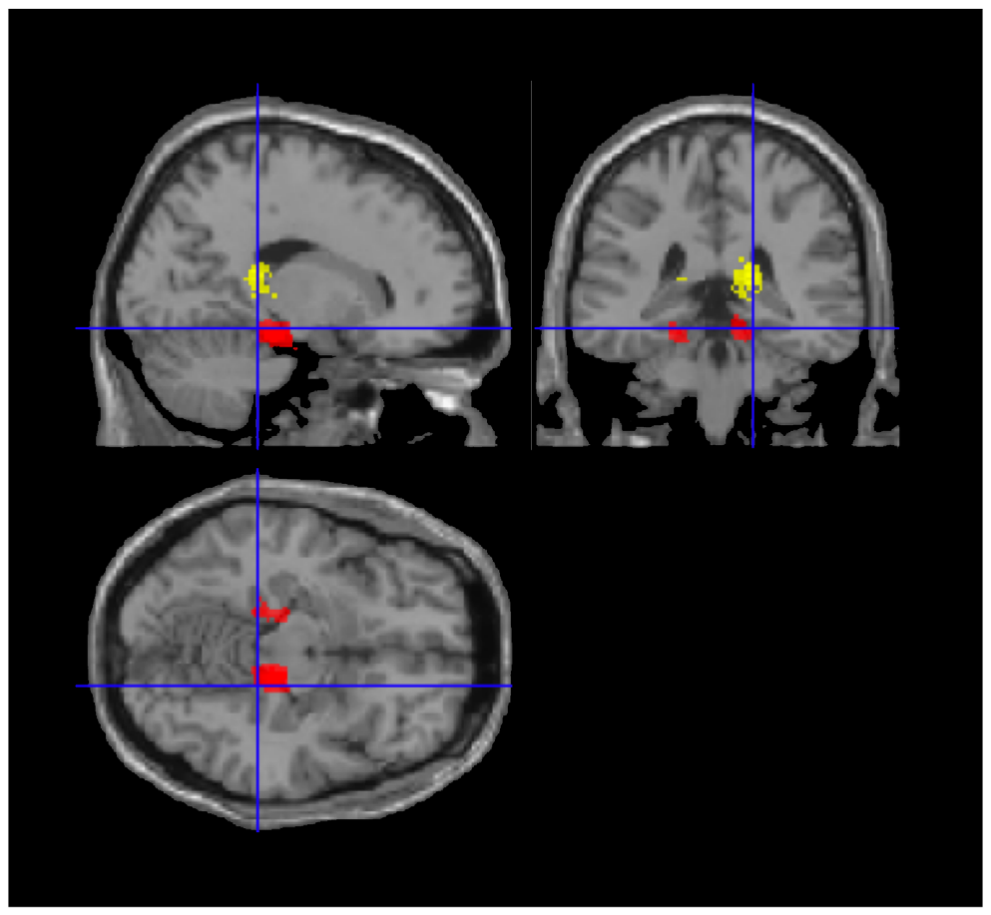
fMRI spatial activation maps for TLE group (yellow) and controls (red) (separate analyses overlaid for comparison). Note the differing location of activation peaks for the identical contrast (task > control). Image coordinates 18, −36, −14.

In the left TLE group, the spatial associative contrast (spatial task > control) revealed a significant cluster of activation in the posterior extent of the right hippocampus proper (Table 3) that peaked in the region of the hippocampal fimbria (Figure 4, left). This included a number of sub-peaks extending into the parahippocampal region. When the peak activation was compared visually with that in the control group (Figure 4, right), the left TLE group's hippocampal activity was also found to be more posterior than the controls' parahippocampal activity. A smaller activation fell within a similar region of the left hemisphere at a more lenient threshold (t_(12)_ = 3.93; p_(uncorrected)_ = 0.001). The left TLE group's performance on the spatial task was compared against the control group's. Activation in the right parahippocampal gyrus was less than that in controls at a threshold just above statistical significance (t_(25)_ = 3.33, p = 0.088).

**Table 3:** fMRI results, left TLE group.

P (cluster corrected)	K (cluster)	T (voxel)	Z (voxel)	Main cluster peak: MNI coords. X, Y, Z	Region
**Spatial task-related activation** *(12 DOF)*					
0.007	172	4.37	3.32	16 −38 16	Right hippocampus (fimbria)
**Spatial task-related deactivation** *(12 DOF)*					
0.186	90	5.27	3.72	40 −56 −6	Right fusiform gyrus
0.003	195	5.01	3.61	−42 −24 54	Left post-central gyrus/parietal lobule
**Spatial-temporal task-related activation** *(10 DOF)*					
0.000	337	5.91	3.79	−10 −38 36	Left posterior cingulate
0.003	168	5.53	3.66	−18 20 40	Left middle frontal gyrus
0.003	173	5.02	3.47	−38 14 26	Left inferior frontal gyrus

Activation contrast: task(s) > matched control. Deactivation contrast: matched control > task(s).

The spatial-temporal contrast in the left TLE group revealed significant task-related activation in the left posterior cingulate region, left middle and inferior frontal gyri (Table 3). Significant task-specific deactivation was not apparent. When examining group differences in patterns of activation and deactivation, while the TLE group did not deactivate any regions to a greater extent than the control group, they demonstrated significantly less deactivation in the right thalamus (t_(23)_ = 3.89, p = 0.013) and a diffuse cluster with peaks in the left hippocampus/PhC and cerebellum (t_(23)_ = 4.21, p = 0.049). At a more lenient threshold additional regions of the posterior left hippocampus and parahippocampal region were deactivated less (t_(23)_ = 3.41; p_(uncorrected)_ = 0.083).

## Discussion

We aimed to examine the role of the parahippocampal cortex (PhC) in elemental spatial and spatial-temporal processing in left MTL impairment. More specifically, we acquired whole brain BOLD data at the time these associations were formed. We used basic visual stimuli (squares and patterns) and selectively increased the spatial and then spatial and temporal information they contained.

In typical adults, highly significant bilateral PhC activation was apparent at the time elemental spatial associations were formed. As the task shifted from spatial to spatial-temporal, there was a change to a task related decrease in joint PhC-hippocampal BOLD signal. Behaviorally, the patients performed at the same level as typical adults, but their performance was supported by memory structures in a different way. During the formation of spatial associations, patients activated the healthy right MTL, showing comparatively greater hippocampal than PhC activity. Consistent with their pathology, however, they failed to activate the left MTL at a standard threshold. In the spatial-temporal task, patients differed from controls in that they did not deactivate the MTL, but instead they engaged parts of a broader extra-MT memory network (posterior cingulate, left prefrontal cortex). The implication of these findings is that the ability to form spatial and spatial-temporal associations in left TLE might be preserved, but that elemental associations are formed with a greater reliance on more extensive networks that include the undamaged (contralateral) mesial temporal region, posterior cingulate and ipsilateral prefrontal cortex, and which therefore reflect neurofunctional reorganization [36]–[38]. This is consistent with the work of Bonelli and colleagues [39] who have demonstrated that in the presence of mesial temporal pathology, memory function engages both the ipsilateral and the contralateral hippocampi. Further work using typical visual and verbal stimuli has been consistent with the contralateral MTL playing a role in compensation for memory function (e.g., [56]). Importantly, other recent and well constructed research has suggested it is engagement of regions within the ipsilateral, pathological rather than contralateral MTL in TLE is associated with better verbal memory performance and that verbal and nonverbal memory systems may respond to mesial temporal damage in different ways [36] [57]. That is, it is no longer clear that verbal task-related activation in the right hippocampus in patients with left TLE represents effective neurocognitive re-organization [36] [38] [40]. In contrast, in relation to the encoding of other stimuli such as simple abstract visual configurations, the right hippocampus might be a key hub of an extensive and diffuse network that supports spatial reorganization [39]. In our data the emergence of right hippocampal activation might be a marker, or an initiator, of the wider network we observed.

In neurocognitive terms, the patterns of recruitment seen in our study might reflect engagement of a flexible ‘*relational*’ representation of elemental associative spatial relationships, rather than a fixed, snapshot-like *associative* representation (for example, [41]). Eichenbaum and colleagues [41] used an altered version of the Morris Water Maze task to show that when the hippocampus, but not the parahippocampal region, was effectively ablated, rats could not navigate a maze using spatial cues if their starting point changed across trials (setting up a demand for relational processing), but were able to navigate the maze if this point was fixed (permitting associative processing). An analogous pattern of impairment is seen in patients with bi-hippocampal damage [42]. Eichenbaum and Bunsey [14] have further argued that relational and associative mechanisms may compete, as evidenced by the finding that damage to a higher-order (relational) component of the system can facilitate function in lower-order (associative) components. In the current task, then, typical adults may use a bilateral associative PhC mechanism for spatial processing and a unilateral hippocampal relational mechanism for the spatial-temporal task. One additional possibility is that this shift from parahippocampal activation to parahippocampal deactivation may also represent a move from basic associative mechanisms to a relational mechanism that also inhibits associative processing. In contrast, left TLE patients with a single functioning MTL may be forced to de-emphasize PhC-mediated associative processing, and instead engage a unilateral, hippocampal domain-general relational mechanism [4]. At an electrophysiological level, rhinal-hippocampal competition might well be underpinned by the inverse rhinal-hippocampal coupling (that is, decoupling) measured in terms of phase synchronization between the two structures. Coupling is modulated by item characteristics, such as word frequency in the case of verbal memory, and appears to be important for successful encoding [43]–[44].

With respect to the observed task-related deactivation, these data contribute to the literature on task-triggered decreases in MTL BOLD signal during associative memory tasks. Raichle [45] describes two possible scenarios in which such relative BOLD ‘deactivation’ occurs. The first is when a brain region is active in both tasks, but less so in the condition of interest (spatial-temporal task) relative to the control. The second occurs when there is a primary reduction in activity in the condition of interest. The first explanation would be consistent with a default-mode type interpretation, but is unlikely here as the (spatial-temporal) task was contrasted with an active control condition. Rather, the current data are consistent with a task-related decrease in BOLD, likely to reflect a decrement in neural firing. Task-related deactivations in relational processing have been demonstrated previously using fMRI. Astur and Constable [46] documented MTL deactivation in relational processing with a transverse patterning task specifically designed to elicit hippocampal activation [47]. Further, Meltzer and colleagues [48] ingeniously demonstrated that deactivation in a memory task reflected a primary reduction in post-stimulus BOLD signal during relational processing. Deactivation did not simply reflect a post-stimulus undershoot, but could be observed in some regions with no initial stimulus peak. BOLD decreases also occur on tasks where decreased neural firing is observed. Cameron et al. [49] recorded directly from human hippocampus and entorhinal cortex (depth electrodes) during paired-associate learning, and observed a majority of hippocampal neurons decreasing their activity during encoding. These decreases reflected subsequent memory: in those hippocampal neurons with encoding-related functional decreases predictive of recall, decreased activity mapped to subsequent recognition and increases to forgetting. Task-related deactivations have also been observed with increasing working memory load (e.g., [58]). As BOLD signal here reflected processes half way through stimulus (spatial) or stimuli (spatial-temporal) presentation, the spatial-temporal deactivation could also be understood as an increase in working memory processes in the spatial-temporal task.

The model of material specificity posits that the left MTL is specialized for verbal mnemonic function and the right for spatial [7]. This view is gradually being refined to incorporate the frequent finding that spatial memory tends to engage the MTL bilaterally (for reviews and evidence see [6], [50]). Glikmann-Johnson et al. [50] showed that on multiple forms of spatial memory, map drawing, navigation, object-location recall, patients with left *or* right hippocampal sclerosis, or anterior temporal lobectomy, were impaired relative to controls. Critically, there was no difference in performance between patients with left or right temporal lobe pathology (see also [51]). In reviewing this and related evidence, Saling [6] concluded that for a measure to effectively tap MTL function in its purest form the measure would need to assess associative processing at an elemental level without cognitive components that recruit higher-order (and lateralized) cognitive constructs. In the current study, task performance did not differ between the patients and controls, suggesting that left mesial temporal damage does not affect elemental forms of spatial encoding, either because this function is not resident in left mesial temporal structures, or because successful reorganization has occurred. Unfortunately, the effects of right hippocampal damage in our tasks could not be tested here. At a neurofunctional level, our data extend previous findings (for example, [39] [52]) to demonstrate that elemental associative processes are also dependent on inter-hemispheric co-operation. Zeidman et al. [30] demonstrated a dissociation between left and right parahippocampal regions and the spatial and spatial frequency characteristics of dot stimuli. They also demonstrated that with appropriate manipulation of fundamental elements of the stimulus and spatial frequency of dots, right-lateralized PhC recruitment occurs. Taken together with our findings and those of others such as Alessio et al. [39] and Treyer et al. [52] this finding illustrates that lateralized activation of the mesial temporal region is most likely to be achieved when the stimulus is pared down to a very fundamental level, and that even quite small increments in complexity begin to recruit bilateral networks. This principle would seem to be fundamental to the design of clinical memory fMRI paradigms [6]. In addition to the theoretical implications of such work, the findings suggest new ways of extending MRI to map brain structure [54] and function [55], potentially through employing a suite of tasks engaging elemental as well as higher-level cognitive functions to resolve the still elusive goal of mapping memory structures presurgically using clinical fMRI [53].

Given the central role of spatial and temporal associative processing in episodic memory [1]–[2], our findings are consistent with the idea that these fundamental contextual components drive MTL engagement in episodic memory, as well as the congeners and derivatives of episodic memory function such as “self projection”, episodic future thought, theory of mind and navigation [16]. Perhaps as a consequence, MTL impairment is associated with deficits in topographic memory (e.g., [17]) and imagination [18] which require the location of oneself in a constructed spatiotemporal context.

### Limitations

In this study, task stimuli were specifically selected as low-level perceptual items that could not be categorized readily as objects. The spatial task was constructed so that successful performance required the perception of a stimulus with significantly more spatial information than a matched control. The relatively anterior location of the observed PhC engagement in the spatial contrast raises the possibility, however, that these stimuli may have been processed as objects, rather than elemental spatial stimuli. While this is possible, and this possibility is difficult to rule out, we believe it is unlikely for a number of reasons. The perceptual complexity and brief display time (0.75s) of the items would have made it difficult for participants to process them as familiar objects; successful task completion could only be achieved by discriminating a viewed stimulus from a lure differing solely in the spatial arrangement of luminance within a random image quadrant. If one of the groups used a verbal strategy to encode stimuli this would also influence the findings; to accommodate this we matched the groups on overall intellectual function (indexed by Matrix Reasoning given patients' left hemisphere pathology). A limitation inherent in designs that seek to examine temporal sequence processing is that increasing temporal information increases working memory load. Because we acquired BOLD information during perception, load may not be a significant issue, but it is important to consider this possibility when interpreting the spatial-temporal findings. An additional limitation was the requirement to image using a sparse acquisition, due to hardware constraints at our center.

### Conclusions

This study demonstrates that left temporal lobe epilepsy changes the neurofunctional substrate of elemental associative processing of spatial and temporal information. It further shows that this functional change occurs in the context of preserved behavioral performance, and therefore can be interpreted as reorganization. Importantly, impairment of left MTL function also alters functioning within the healthy right MTL where, in contrast to typical adults, posterior hippocampus is engaged in concert with the parahippocampal region during spatial associative processing. In more complex spatial-temporal associative processing, patients with TLE then rely additionally on extra-hippocampal MTL structures.
